# A Theory-Guided Machine Learning and Molecular Dynamics Approach for Characterizing Fast-Curing Polyurethane Systems

**DOI:** 10.3390/polym18060679

**Published:** 2026-03-11

**Authors:** Luohaoran Wang, Jacob Harris, Steven Mamolo, Sangharsha Gharat, Ali Zolali, Alan Taub, Mihaela Banu

**Affiliations:** 1 Department of Mechanical Engineering, University of Michigan, Ann Arbor, MI 48109, USA; lhrwang@umich.edu (L.W.); sgharat@umich.edu (S.G.); alantaub@umich.edu (A.T.); mbanu@umich.edu (M.B.); 2 Department of Aerospace Engineering, University of Michigan, Ann Arbor, MI 48109, USA; smamolo@umich.edu; 3 BASF Corporation, Wyandotte, MI 48192, USA; ali.zolali@basf.com; 4 Department of Material Science and Engineering, University of Michigan, Ann Arbor, MI 48109, USA

**Keywords:** polyurethane, cure kinetics, differential scanning calorimetry (DSC), gelation, glass transition temperature (*T_g_*), degree of cure (*DoC*), molecular dynamics (MD)

## Abstract

Fast-curing polyurethane (PU) systems are attractive for high-throughput manufacturing, but quantifying cure kinetics, gelation, and cure-dependent glass transition temperature ($T_{g}$) is difficult, especially at a low degree of cure (*DoC*). Here, a fast-reacting BASF PU formulation was studied using non-isothermal differential scanning calorimetry (DSC) at multiple heating rates, rheometry at 50 °C, and molecular dynamics (MD) simulations to extend $T_{g}\left(\alpha \right)\;$in the low-*DoC* regime. DSC provided reaction enthalpy and conversion histories, and Kamal–Sourour (KS) parameters were identified by robust nonlinear fitting, reproducing conversion and curing rate profiles (*R*^2^ > 0.99 and >0.95). Rheology indicated gelation between 475 and 625 s (*DoC* ≈ 0.53), and DSC-based $T_{g}$ at uncured, gelation, and fully cured states, established the experimental $T_{g}$ trend. MD (LAMMPS) with topological crosslinking and NPT thermal scans extracted $T_{g}\;$from density–temperature slopes at selected *DoC* points. Experimental and MD $T_{g}$ data were fused with Gaussian process regression constrained by the DiBenedetto relationship (5-fold cross-validation), giving *λ* ≈ 0.29 and confidence intervals. This framework links kinetics, gelation, and $T_{g}$ evolution for fast-curing PU and identifies the low-*DoC* region as the main source of uncertainty.

## 1. Introduction

Polyurethane (PU) is a thermosetting polymer formed by the reaction of isocyanates and polyols, characterized by a segmented molecular structure consisting of soft segments and hard urethane bonds [1]. The chemical structure of polymers, together with their processing methods, ultimately governs the macroscopic properties of the material, thereby determining its suitability for downstream functional applications [2]. Owing to the versatility of their chemistry, PU can be synthesized from a wide range of structural units with varying molecular sizes and chemical functionalities. This flexibility enables the design and fabrication of a diverse family of PU materials with tailored chemical architectures, allowing their mechanical and thermal properties to be systematically tuned to meet specific application requirements [3]. PU is ubiquitous in structural, automotive, and aerospace applications, including insulators, foams, and binders [1,4]. Pultrusion is a continuous manufacturing process widely used to produce fiber-reinforced polymer composites [5]. Among thermoset polymers, PU has emerged as a preferred matrix material due to its rapid curing kinetics and superior mechanical performance, making it highly suitable for pultruded applications in aviation, transportation, and construction [6,7]. Despite its technological importance, quantitatively tracking the transition from a low-viscosity liquid to a gel and ultimately a solid during curing remains challenging, particularly for the glass transition behavior. Experimental characterization techniques, such as differential scanning calorimetry (DSC) and thermomechanical analysis (TMA), are widely used to measure curing heat flow and thermomechanical responses, such as thermal expansion, respectively [8,9,10]. However, for fast-curing PU, accurately capturing $T_{g}$ at low degrees of cure is difficult because rapid kinetics and overlapping thermal signals limit experimental resolution in the early curing regime. On the other hand, $T_{g}$ is not governed by a fixed cure degree but rather by the attainment of a critical network density, *ν_e_*, corresponding to the formation of an infinite molecular network [11]. Current industrial practice often assumes $T_{g}$ and gelation as a fixed material property or directly correlates it with a single degree of cure (*DoC*), α, which oversimplifies the physical mechanisms governing network evolution and segment migration during curing [11,12]. This can lead to inaccurate predictions of $T_{g}$ and gelation onset, resulting in defects such as resin-rich zones, incomplete fiber wet-out, or premature solidification. A more fundamental, physics-based description of polymerization is needed to improve process control and reproducibility. Recent reviews of polyurethane (PU) cure kinetics characterization emphasize that calorimetry/spectroscopy alone is often insufficient and that rheological monitoring is essential for capturing evolving physical properties (e.g., viscosity, gelation, pot life), particularly once diffusion and heterogeneity become important [13]. Related multi-technique characterization and modeling workflows have also been demonstrated for fast-curing PU adhesives to link conversion to thermomechanical transitions and processing windows [14].

Molecular dynamics (MD) simulation provides a molecular-level prediction of glass transition behavior from an atomistic structure and thermomechanical response. According to Han et al. [15], the $T_{g}$ of polyethylene, polystyrene, and polyisobutylene was captured by evaluating the thermal expansion of each polymer, and $T_{g}$ was defined at the intersection point of lower temperatures and higher temperatures. In addition, the Enhanced Monte Carlo (EMC) package developed by Pieter J. in-’t Veld [16] has been used to generate equilibrated polymer configurations and structural descriptors prior to thermomechanical analysis. In fast-curing PU, these simulation and experimental datasets are often sparse and noisy in the low-*DoC* regime, motivating data fusion approaches that enforce physical consistency. Recent advances in machine learning (ML) provide a powerful pathway to address these challenges. Probabilistic surrogate models, particularly Gaussian process regression (GPR), are well suited for learning smooth structure–property relationships from sparse and noisy datasets while simultaneously providing quantified predictive uncertainty [17,18]. When coupled with physics-informed priors, such as curing kinetics or thermodynamic constraints, GPR-based surrogates can further guide MD simulations and experimental design, enabling more efficient exploration of the parameter space and targeted data acquisition. In this work, MD simulations with topological crosslinking and thermal expansion analysis were used to estimate crosslink density evolution and $T_{g}$ across degrees of cure [19,20,21,22]. These results were integrated with experimental characterization to construct a conversion-dependent transition model and an uncertainty-aware predictive $T_{g}$*-(α)* relationship using theory-guided machine learning. The resulting framework supports process-modeling efforts for fast-curing PU systems incorporated in pultrusion. Similar MD-based determinations of $T_{g}$ and the volumetric coefficient of thermal expansion (CTE) from density/specific volume–temperature trends have been reported for thermoset polymer nanocomposites [23]. Hybrid all-atom/coarse-grained MD strategies have further been used to connect microphase morphology and hydrogen bonding to macroscopic toughness in related segmented urea-based elastomers (polyureas), illustrating the value of multiscale simulation for urethane/urea chemistries [24].

Unlike conventional industrial approaches that assign a single glass transition temperature to a specific *DoC*, the proposed framework constructs a continuous and uncertainty-aware *T_g_*–*DoC* relationship by integrating experimental measurements with molecular-scale simulations. This hybrid strategy enables physically consistent extrapolation into low-conversion regimes where experimental resolution is limited, thereby improving predictive reliability across the full curing range.

## 2. Materials and Methods

### 2.1. Material

In this study, ELASTOCOAT^®^ 74850R resin and ELASTOCOAT^®^ 74850T isocyanate were supplied by BASF North America, Wyandotte, MI, USA. These two components were used as received without further purification and mixed according to the manufacturer-recommended formulation. ELASTOCOAT^®^ 74850R serves as the polyol component, while ELASTOCOAT^®^ 74850T acts as the reactive isocyanate, together forming a fast-curing polyurethane system suitable for curing kinetics investigations.

#### 2.1.1. Curing Kinetics Evolution

To evaluate the curing kinetics model for PU, dynamic DSC measurements were applied with Discovery DSC 2500 from TA, New Castle, DE, USA. Polyol and isocyanate were stored in an insulated container with freezer packs to maintain a temperature of around 5 °C. Plastic film and zip bags were used to prevent moisture from the environment. Since the PU system is known to react even at subzero conditions (initiating as low as −25 °C), these precautions slowed down the reaction kinetics and ensured that minimal curing occurred prior to DSC measurement. Thus, the recorded exothermic heat accurately reflects the intended curing process. Non-isothermal DSC conducted at multiple heating rates, combined with isoconversional and model fitting approaches, is a common strategy for identifying apparent activation energies and multi-step mechanisms in fast-curing PU systems [25].

To investigate the dynamic curing kinetics of the PU system, the samples were subjected to a non-isothermal heating program over the temperature range of −50–250 °C at a constant heating rate of 3, 5, 10, and 20 °C/min.

#### 2.1.2. Gelation

A TA Instruments Discovery HR30 rheometer was used to measure the viscosity evolution of the PU system using dynamic oscillatory tests. To correlate viscosity with the *DoC*, experiments were conducted at 50 °C under various angular frequencies. The evolution of curing behavior was monitored to identify the gelation point. All tests were performed using 20 mm diameter parallel plates with a gap of 300 microns. Rheometry is particularly valuable for PU systems because it directly captures physical property evolution during cure; for heterogeneous reactions, these physical changes can dominate and are not fully accessible through thermal or spectroscopic techniques alone [13].

#### 2.1.3. Glass Transition Temperatures

To capture the glass transition temperature, the following isothermal and dynamic scanning DSC method was used. Partial curing of the PU samples was achieved through isothermal holding at 100 °C for durations of 3, 5, 15, and 30 min, respectively. Cooling down to −60 °C, holding 5 min, and ramping to 250 °C with 10 °C/min were performed. Under the same measure groups and testing conditions, the exothermic behavior during the cooling steps was neglected. $T_{g}\;$was determined by DSC from the second heating cycle when a distinguishable glass transition was observed. At low degrees of cure (below 0.5), $T_{g}\;$could not be reliably resolved due to weak or overlapping thermal transitions.

#### 2.1.4. Molecular Dynamic Simulation Capturing *T_g_*

Due to the limited understanding of glass transition behavior below the 0.5 *DoC*, accurately predicting $T_{g}$ evolution in the early curing regime remains challenging. MD simulations can be employed to help bridge this gap by providing molecular-level insight into network formation and thermomechanical behavior at low *DoC*.

A PU MD model was set up in LAMMPS [26] with a 50.00 × 50.00 × 50.00 Å^3^ cubic simulation box and a density of 1120 kg/m^3^. The optimized potentials for the liquid simulation all-atom (OPLS-AA) force field were used to define the interaction between atoms for the crosslinking simulation. Time integration was performed using the velocity Verlet algorithm with a time step of 0.25 fs. Long-range electrostatic interactions were computed using the particle–particle particle–mesh (PPPM) method, with a cutoff distance of 1.2 nm [22]. In addition, “Condensed-phase Optimized Molecular Potential for Atomistic Simulation Studies” (COMPASS) and “Polymer consistent force field” (PCFF) were applied to investigate the volume expansion capturing $T_{g}$ [27,28].

**Figure 1:**
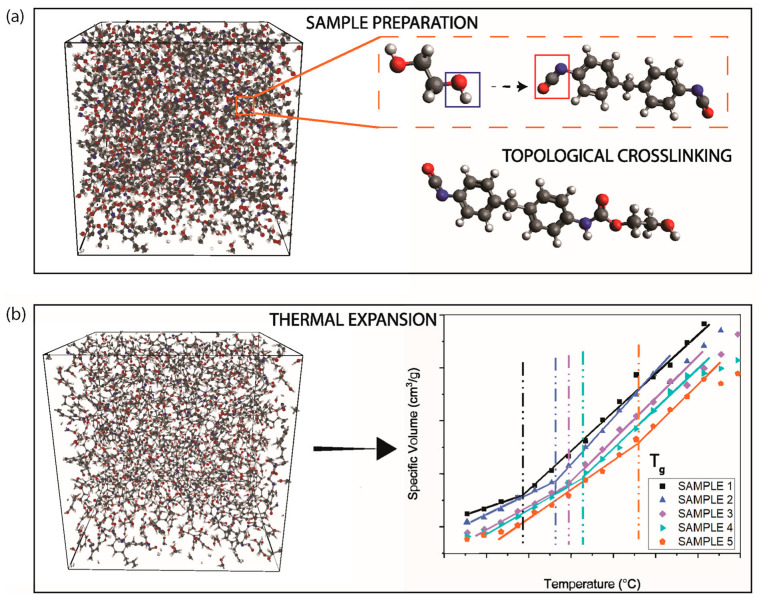
MD simulation workflow for PU network formation and *T_g_* determination: (**a**) sample preparation and topological crosslinking; (**b**) thermal expansion analysis to obtain *T_g_* from specific volume–temperature curves.

A model of 400 monomers was used in the simulation box, corresponding to a polyol-to–isocyanate weight ratio of 46:54, as provided by BASF. The number of monomers was determined based on their molecular weights, and the resulting simulation configuration is shown in Figure 1. To enable crosslinking between the target reactive functional groups and to capture the $T_{g}$ of the polymer system, four sequential simulation steps were implemented in LAMMPS, such as constant number of particles, pressure, and temperature (NPT) densification, crosslinking, creating chemical bonds, and *T_g_* scanning. During NPT densification, the environment was equilibrated at 300 K under 1atm pressure. After densifying the simulation box, the crosslinking in this work was introduced using a topology algorithm without relying on chemical reactions. The hydroxyl oxygen atoms in the polyol and the double-bonded carbon atoms in the isocyanate were identified as active atoms. The crosslinks were formed when the distance between active atoms was equal to or less than the cutoff radius of 3.0 Å [22], and the schematic of this behavior is presented in Figure 1. The chemical bonds between atoms were introduced based on the parameter data file generated from the EMC package. $T_{g}\;$scanning was performed after chemical bonds mapping. First, the crosslinked PU system was loaded along with force field parameters, and the initial structure underwent energy minimization to eliminate unreasonable contacts. Subsequently, isothermal stabilization is performed under NPT conditions at high temperature to fully homogenize the system and erase initial configuration history. Based on this, the external pressure is held constant while the system temperature is linearly decreased from $T_{high}$ to $T_{low}$ under NPT conditions to obtain the density–temperature response during cooling. A brief isothermal NPT stabilization at the low-temperature end minimizes nonequilibrium effects. Finally, under identical pressure conditions, the temperature is linearly raised from $T_{low}\;$to *T_high_* to derive the density vs. temperature curve during heating. $T_{g}\;$was determined by segmented linear fitting of the density vs. temperature curves from the cooling and heating phases, with the intersection point of the two segments’ slope changes serving as the $T_{g}$ value.

### 2.2. Physics-Informed Prediction

To predict the glass transition behavior of the PU system based on experimental DSC data and MD simulation results, GPR was employed as a bridging framework to fit the target *T_g_* curve [17,18]. In this approach, *T_g_* (*α*) data obtained from experiments and MD simulations were used as training inputs, guided by the physically motivated DiBenedetto equation as a prior constraint.

The DiBenedetto relationship is given by Equation (1) [29]:

(1) $$T_{g}\left(\alpha \right)=T_{g0}+\left(T_{g\infty }-T_{g0}\right)\frac{\lambda \alpha }{1-\left(1-\lambda \right)\alpha }$$

The outputs of this model include the DiBenedetto parameter (*λ*), a continuous *T_g—_α* relationship, and the associated data uncertainty. To enable smooth and continuous material property prediction, a Matérn kernel was selected [30]. A constant term was introduced to provide additional flexibility, and the kernel was combined with a noise term to account for random noise arising from experimental measurements and MD fluctuations [31]. The Matérn kernel function can be expressed as in Equation (2), and the final kernel function is presented by Equation (3):

(2) $$k\left(x_{i},x_{j}\right)=\frac{1}{\Gamma \left(v\right)2^{v-1}}\left(\frac{\sqrt{2v}}{l}d\left(x_{i},x_{j}\right)\right)XK_{v}(\frac{\sqrt{2v}}{l}d\left(x_{i},x_{j}\right))\;$$

where *d*(*x_i_, x_j_*) is the Euclidean distance, *K_v_* is a modified Bessel function, *ε* is noise, Γ is the gamma function, v is the smoothness parameter controlling the differentiability of the Matérn kernel, and l is the length scale parameter.

(3) $$Kernel=C\cdot k\left(x_{i},x_{j}\right)+\varepsilon (\sigma_{n}^{2}\delta_{ij})$$

where *C* is a constant. The term $\varepsilon \left(\sigma_{n}^{2}\delta_{ij}\right)\;$represents the observation noise variance added to the diagonal of the covariance matrix; $\delta_{ij\;}$ is the Kronecker delta, equal to 1 when i=j and 0 otherwise.

As this study relies on hybrid data sources with a limited number of data points, cross-validation is critical to ensure robust and reliable predictions. Therefore, *k*-fold cross-validation [32] was applied to evaluate model performance and mitigate overfitting. Within this framework, the relationship between the observed data and the latent target function can be expressed as:

(4) $$Target:T_{g}\left(\alpha \right)=T_{g0}^{DiB}\left(\alpha ;\lambda \right)+\delta (\alpha )$$

(5) $$T_{g,i}^{obs}\left(\alpha_{i}\right)=T_{g}\left(\alpha_{i}\right)+\varepsilon_{i}\;\varepsilon_{i}\sim N(0,\;\sigma_{i}^{2})$$

(6) $$\sigma_{i}^{2}=\left\{\begin{array}{c} \sigma_{Exp}^{2},\\ \sigma_{MD}^{2}, \end{array}\;\begin{array}{c} i\in Experiment\\ i\in MD \end{array}\right.$$

where $\sigma_{Exp}^{2}$ and $\sigma_{MD}^{2}$ represent the noise variances associated with experimental and MD data points, respectively; δ(α) is the residual correction.

A constant experimental noise level *σ_Exp_* = 0.4 was assumed, while the MD noise level *σ_MD_* was automatically selected by minimizing a predefined objective function. The fitting performance was evaluated using the root mean square error (*RMSE*), defined as

(7) $$RMSE_{CV}\left(\lambda ,\sigma_{MD}\right)={\left[\frac{1}{N}\sum_{k=1}^{K}\sum_{i\in D_{val}^{\left(k\right)}}{\left(T_{g,i}^{obs}-T_{g}^{\left(k\right)}\left(\alpha_{i}\right)\right)}^{2}\right]}^{\frac{1}{2}}$$

where *D_val_*^(*k*)^ denotes the validation subset in the *k*-th fold during the *k*-fold cross-validation procedure; K is the number of cross-validation folds; *N* is the total number of validation data points across all folds; $T_{g,i}^{obs}$ is the observed glass transition temperature of the i-th data point; and $T_{g}^{\left(k\right)}(\alpha_{i})$ is the predicted glass transition temperature evaluated using the model trained in the k-th fold at the degree of cure $\alpha_{i}$.

In addition, a curvature regularization term $J_{curv}$ was introduced to discourage spurious oscillations in the predicted $T_{g}$*–DoC* relationship. Since the true evolution of $T_{g}$ with curing was expected to be smooth and physically monotonic, model selection was performed by minimizing the cross-validated *RMSE* together with the curvature regularization term, and the target function is described in Equation (8) [33,34,35].

(8) $$S\left(\lambda ,\sigma_{MD}\right)=\arg\min\left[RMSE_{CV}\left(\lambda ,\sigma_{MD}\right)+J_{curv}\right]$$

The Matérn kernel hyperparameters were determined using a constrained grid search over physically reasonable ranges of the smoothness parameter ν ∈ [0.3, 1.5] and length scale l ∈ [0.1, 3.0]. Rather than performing an exhaustive global optimization, a localized sweep was conducted over these intervals. The optimal configuration was selected by minimizing a composite objective function combining cross-validated RMSE with a curvature-based regularization term to suppress non-physical oscillations in the predicted response.

The final selected values were *ν* = 0.58 and *ℓ* = 2.17, with *σ_MD_* = 0.4. The relatively large length scale indicates that the *T_g_*–*DoC* relationship exhibits globally smooth behavior over the narrow *DoC* interval considered in this study. This physics-informed hyperparameter selection strategy ensures stable surrogate behavior while maintaining predictive accuracy within the experimentally accessible *DoC* range.

## 3. Results

### 3.1. Curing Kinetics Evolution

**Figure 2:**
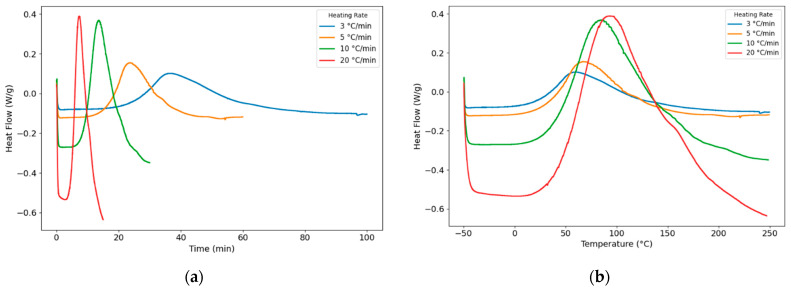
Dynamic heat flow results of PU within (**a**) time; (**b**) temperature under 3, 5, 10, and 20 °C/min heating rates.

To capture the first glass transition temperature, $T_{g,0}$, a PU sample was tested from −80 to 250 °C and back at 5 °C/min. The initial glass transition temperature, $T_{g,0}$, was −56.32 °C, and the final glass transition temperature, $T_{g,\infty }$, was 89.12 °C. Figure 2 presents the normalized heat flow curves of the PU system. As shown in Figure 2b, the dynamic curing processes under three different heating rates were recorded and analyzed using DSC. The corresponding curing characteristic parameters are summarized in Table 1, including the onset curing temperature (*T_onset_*), peak temperature ($T_{p}$), endset curing temperature (*T_endset_*), and the overall curing temperature range of the PU system. The total reaction enthalpy ($∆H_{tot}$) was determined by integrating the area under the exothermic peak in Figure 2a according to Equation (9), and the average value was around 138.10 J/g.

(9) $$∆H_{tot}=\int_{onset}^{endset}\dot{q}\left(t\right)dt$$

where $\Delta H_{\mathrm{tot}}$ is the total reaction enthalpy, $\dot{q}(t)$ is the DSC heat flow rate as a function of time, and $T_{onset}$ and $T_{end}$ denote the onset and endset temperatures of the curing reaction, respectively.

**Figure 3:**
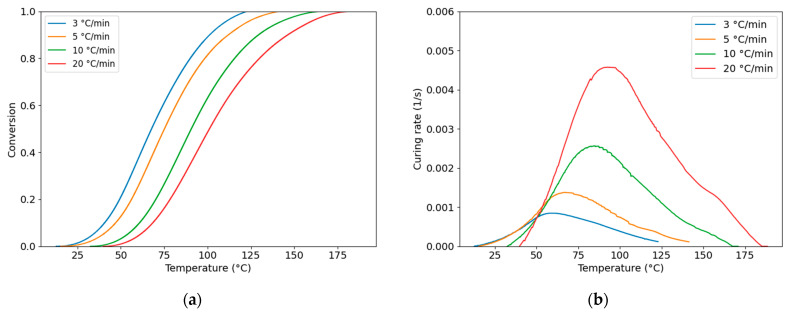
(**a**) Experimentally determined degree of cure of PU; (**b**) curing rate curves for PU under 3, 5, 10, and 20 °C/min heating rates.

To evaluate the conversion of the PU system, the degree of cure α and the curing rate $\frac{d\alpha }{dt}$ were integrated based on Equations (10)–(12) [36]. The conversion and curing-rate curves were calculated and are plotted in Figure 3a and Figure 3b, respectively, for different heating rates.

(10) $$\alpha =\frac{∆H}{∆H_{tot}}$$

(11) $$∆H=\int_{t_{0}}^{t}\dot{q}\left(t\right)dt$$

(12) $$\frac{d\alpha }{dt}=\frac{\dot{q}\left(t\right)}{∆H_{tot}}$$

The curing rate can be evaluated in kinetic analysis, and the kinetic process can be described as [37]

(13) $$\frac{d\alpha }{dt}=\frac{\dot{q}\left(t\right)}{∆H_{tot}}=K\left(T\right)f(\alpha )$$

where $\dot{q}$ is heat flow during the DSC test; *K*(*T*), as a temperature-dependent reaction rate constant, is described by Equation (14); and *f*(α) is a dependent kinetic model function that can be calculated using the logarithmic form of the kinetics below

(14) $$K\left(T\right)=Aexp(-\frac{E_{a}}{RT})$$

(15) $$ln\frac{d\alpha }{dt}=\ln\left[Af\left(\alpha \right)\right]-\frac{E_{a}}{RT}$$

where A is the pre-exponential factor; R is the universal gas constant.

**Figure 4:**
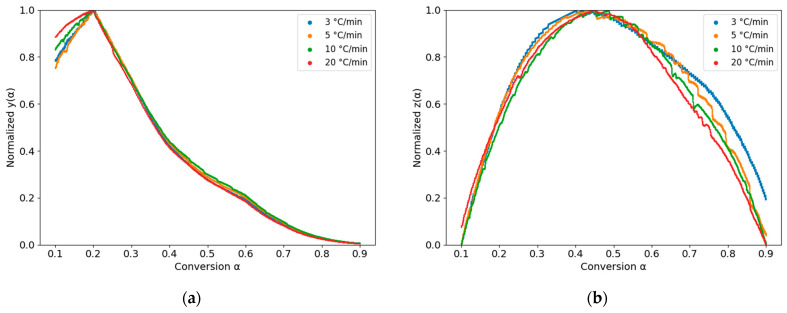
Variations of (**a**) *y*(α) and (**b**) *z*(α) functions vs. conversion for the PU system.

The activation energy ($E_{a}$), a key parameter in the curing kinetics model, was determined from the slope of the corresponding linearized isoconversional equation. To improve numerical stability and accuracy, the experimental data were smoothed and normalized by introducing the auxiliary functions y(α) and z(α) [38,39]. The curves of these functions are presented in Figure 4. Furthermore, the temperature integral is approximated using the fourth-order rational expression, $\pi \left(x\right)$, proposed by Senum and Yang [40].

(16) $$y\left(\alpha \right)=\left(\frac{d\alpha }{dt}\right)e^{x}$$

(17) $$z\left(\alpha \right)=\pi \left(x\right)\left(\frac{d\alpha }{dt}\right)\frac{T}{\beta }$$

where β is the DSC heating rate.

The pre-exponential factor, A, can be determined by Equation (18) [37,41]

(18) $$A=\left(\frac{\beta x_{p}}{Tf’(\alpha_{p})}\right)exp\;x_{p}$$

where $f^{\prime }(\alpha_{p})$ is the derivative of the kinetic model with respect to the degree of cure, $\alpha_{p}$ is the degree of cure corresponding to the maximum reaction rate on the DSC curve, the subscript p denotes quantities evaluated at the peak of the DSC exotherm, and $x_{p}$ denotes the reduced activation energy, defined as $x_{p}=E_{a}/(RT_{p})$, with $T_{p}$ corresponding to the peak temperature of the DSC curve.

The maximum values of $\alpha_{p}$,$\;\alpha_{m}$, and ${\alpha_{p}}_{\infty }$ obtained, respectively, from the curing rate curve, *y*(*α*), and *z*(*α*) functions, are summarized in Table 2. According to [38], the autocatalytic model with two reacting factors, *m* and *n*, is applicable when ${\alpha_{p}}_{\infty }$ < 0.632. To account for both autocatalytic and non-autocatalytic reaction pathways, the Kamal–Sourour (KS) kinetic model was employed [42]. The KS model can be expressed as follows:

(19) $$\frac{d\alpha }{dt}=[k_{1}+k_{2}\alpha^{m}]{\left(1-\alpha \right)}^{n}$$

where $k_{1}$ and $k_{2}$ represent the rate constants associated with the chemical-controlled and autocatalytic reactions, respectively, m denotes the autocatalytic reaction order, and n represents the overall reaction order with respect to the unreacted fraction.

Based on the global optimizer, the KS kinetic parameters were determined through robust nonlinear least squares fitting using a soft L1 loss function [43]. This approach mitigates the impact of experimental noise in the DSC data, particularly at the extremities of the curves, resulting in stable and physically meaningful parameter estimates. Due to the rapid curing behavior of the PU system, the dataset collected at the highest heating rate was excluded from the fitting. To ensure computational stability and avoid negative values, the parameters were log-transformed. The robust loss optimization function was employed to minimize the residuals, with the objective function described using the soft L1 loss function, as defined below:

(20) $$r_{i}=w_{i}[\alpha_{pred}\left(A_{1},\;A_{2},\;m,\;n,\;E_{a}\right)-\alpha_{exp}]$$

(21) $$\min F=\sum_{i}\rho \left(\frac{r_{i}}{\sigma }\right)$$

where $r_{i}$ denotes the residual of the i-th data point, $\sigma_{i}$ is the corresponding noise scale, and ρ is a robust loss function introduced to reduce the influence of outliers.

**Figure 5:**
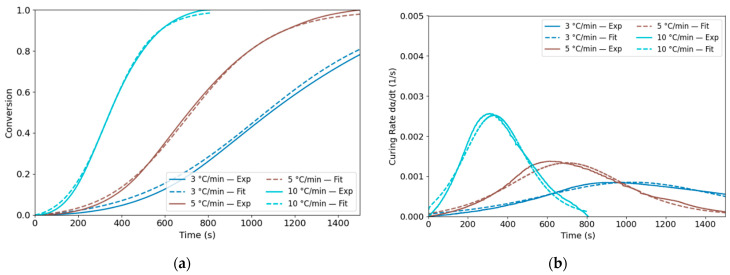
Fitted curves of (**a**) degree of cure and (**b**) curing rate for the PU system.

The fitting curves are shown in Figure 5 and compared with the experimental data. The fitted *DoC* curves closely matched the experimental results, with $R^{2}$ values listed in Table 3, all exceeding 0.99. Overall, the $R^{2}$ values for the curing rate were consistently greater than 0.95, indicating high accuracy.

Based on the KS equation and fitted curves, the critical values of the KS kinetics curing model are listed in Table 4.

### 3.2. Gelation

Time sweep oscillation tests were conducted at 50 °C with angular frequencies (ω) of 1, 3, 10, 30, and 100 rad/s. Gel time can be estimated by measuring low-amplitude oscillatory shear and tracking changes in storage modulus ($G^{\prime }$), loss modulus ($G^{\prime \prime }$), and complex viscosity ($\eta^{*}$). To identify the gelation point for the PU system, three methods were considered: the crossover point between storage and loss modulus, the frequency-independent intersection of the dissipation factor $\left(\tan\left(\delta \right)=\frac{G^{\prime \prime }}{G^{\prime }}\right)$, and the moment when viscosity becomes practically infinite [44]. For a typical thermoset, viscous behavior dominates in the early stages of curing ($G^{\prime \prime }$ > $G^{\prime }$). As the degree of crosslinking increases, there is often a rapid rise in elasticity, which begins to dominate the system ($G^{\prime }$ > $G^{\prime \prime }$). The intersection, $G^{\prime }$ = $G^{\prime \prime }$, is taken as the apparent gel point [45,46]. In some cases, no clear intersection point is observed within the measurement time window. This may be attributed to the characteristic timescale $\left(\tau =\frac{1}{\omega }\right)$, which determines how long the material has to respond within each oscillation cycle (i.e., a shorter timescale means the material is “probed” more frequently). At low angular frequencies, the large timescale enables polymer chain relaxation, allowing the loss modulus to dominate longer into the cure. In such cases, gelation can be assessed using criteria based on the dissipation factor or complex viscosity. Of the five angular frequencies tested, crossover between the storage and loss modulus occurred only during the 100 rad/s trial, at approximately 200 s. This is not taken to be the gel point—rather, it is attributed to the very short deformation timescale (τ=0.01 s), resulting in mildly elastic-dominant behavior as the polymer network rapidly began forming during the early stages of curing.

**Figure 6:**
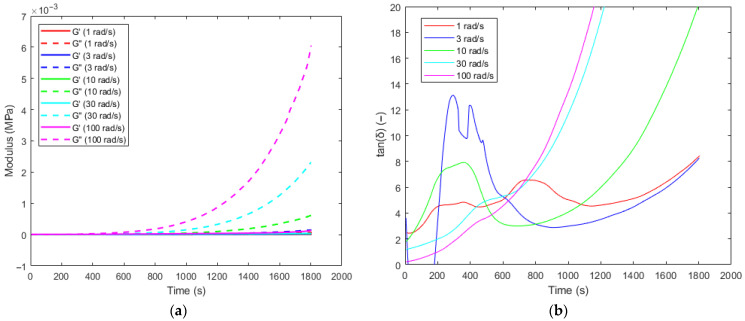

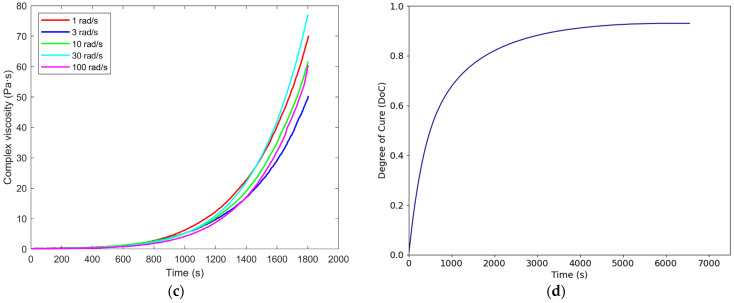
(**a**) Storage and loss modulus; (**b**) dissipation factor; (**c**) complex viscosity versus time at angular frequencies of 1, 3, 10, 30, and 100 rad/s and a temperature of 50 °C; (**d**) *DoC* versus time at 50 °C isothermally from DSC.

During the first 400–500 s of curing, the low magnitudes and close proximities of the storage and loss moduli in Figure 6a, coupled with experimental noise, led to significant instability in the dissipation factor for all trials. Thus, when computing $\tan\left(\delta \right)$ for the curves in Figure 6b, $G^{\prime }$ and $G^{\prime \prime }$ were first smoothed using a third-order Savitzky–Golay filter with a frame length of 21. Winter–Chambon theory states that, at the gel point, the storage and loss moduli grow proportionally as a function of frequency [47]. Therefore, the gel point can be identified by determining when the dissipation factor becomes independent of angular frequency. In Figure 6b, the most notable intersection occurs between 600 and 625 s, for which four of the five curves intersect. The 10 rad/s trial intersects most of the other curves slightly earlier, between 475 and 550 s. Thus, the $\tan\left(\delta \right)$ crossover method indicates a gelation window of 475–625 s, during which the true gel point is likely to exist.

The final method for determining the gel point is based on the premise that fully crosslinked networks exhibit a complex viscosity that diverges toward infinity. This method often relies on visual cues to identify the gel point, such as spikes or plateaus across various frequencies. No obvious features are present in Figure 6c to indicate the gel point via this method—in linear or log scale. When mapping the selected points to the *DoC* curve under 50 °C in Figure 6d, the gelation point occurred around *DoC* = 0.53.

### 3.3. Glass Transition Temperatures

**Figure 7:**
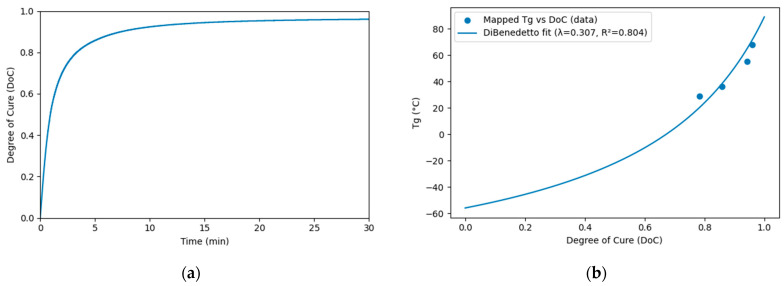
(**a**) Conversion curve; (**b**) *T_g_* fitting curve of PU under isothermal conditions at 100 °C.

Three glass transition temperatures, $T_{g0}$, $T_{g,gel}$, and $T_{g\infty }$, were measured for uncured resin, gelation point, and cured resin, respectively. To determine $T_{g}$ as a function of conversion $\left(\alpha \right)$ for a fast-curing PU system, an integrated approach was employed [10]. The procedure combines isothermal curing within a DSC, rapid quenching, and subsequent dynamic scanning. The conversion $\left(\alpha \right)$ was calculated based on the residual enthalpy measured during the DSC analysis. All time references were synchronized to the DSC cell clock, effectively eliminating uncertainties related to sample preparation. The isothermal DSC test was conducted at 100 °C for 30 min, and the corresponding conversion curve is shown in Figure 7a. The PU system reached full cure at approximately 30 min under isothermal conditions. The time–temperature–transformation (TTT) diagram was constructed based on the Di Benedetto equation. The fitted glass transition temperature as a function of time is shown in Figure 7b, with the fitted constant λ of approximately 0.31. Similar calorimetry-based $T_{g}(\alpha )$ characterization and construction of TTT-type processing diagrams have been reported for fast-curing polyurethane adhesives to support model calibration and process window definition [14].

### 3.4. Molecular Dynamics Simulation Capturing T_g_

**Figure 8:**
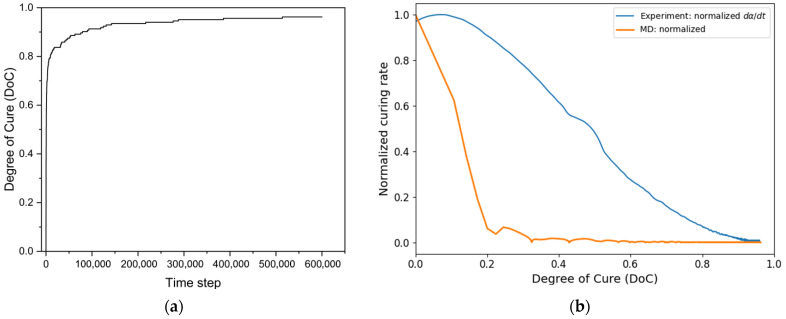

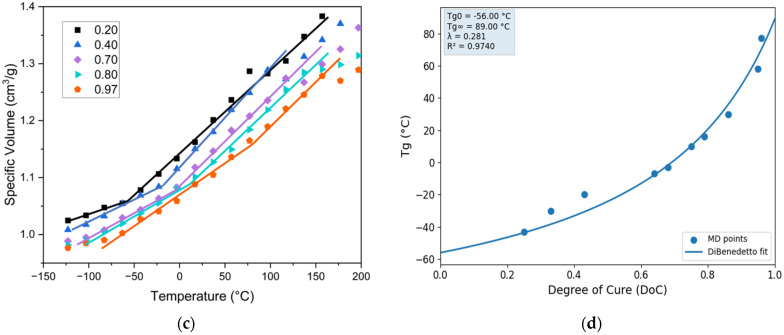
(**a**) Conversion curve from MD simulations under isothermal conditions at 100 °C; (**b**) comparison of curing rates between experimental measurements and MD simulations; (**c**) thermal expansion measurement with different *DoC* models; (**d**) fitted DiBenedetto curve from MD simulations.

To compare the MD model with the experiment, the unreactive PU system was heated to 100 °C, as previously described. The *DoC* vs. step time curve is plotted in Figure 8a. Crosslinking was rapid in the beginning, followed by a gradual slowing until it reached 0 due to the limit of the reactive functional groups. The curing rate from the MD simulation was normalized and compared to the curing rate with the experimental curve, which is shown in Figure 8b. The curing rates were mapped to each *DoC* point. The experimental curve shows a higher reaction rate at the beginning of the reaction compared to the MD curve, which shows that the isocyanate–hydroxyl reaction is highly reactive, with reaction sites achieving instantaneous and complete contact [48,49]. By comparing the curing rate trends of the two groups, it was observed that chemical reactions dominate the crosslinking process during curing. To evaluate the $T_{g}$ of the MD model, 10 *DoC* points were selected: 0.2, 0.3, 0.4, 0.65, 0.7, 0.75, 0.8, 0.85, 0.95, and 0.97. $T_{g}$ was determined from the heating-induced specific volume temperature curves by identifying the intersection of the linear fits to the glassy and rubbery regimes, and five curves of different *DoC* models are presented in Figure 8c. As the degree of cure increases, the specific volume of the polymer systematically decreases, reflecting the progressive formation of a denser crosslinked network and the reduction in free volume.

The resulting $T_{g}$–α relationship is shown in Figure 8d. The extracted $T_{g}$ data were fitted using the DiBenedetto equation, with the initial and fully cured $T_{g}$ values fixed. The fitted curve shows excellent agreement with the MD data, yielding a high coefficient of determination ($R^{2}=0.97$), indicating that the DiBenedetto model effectively captures the evolution of $T_{g}$ with *DoC*.

A GPR-based predictive framework was implemented to robustly model the DiBenedetto relationship while explicitly accounting for experimental and MD noise through additive noise terms.

### 3.5. Gaussian Process Regression Prediction

**Figure 9:**
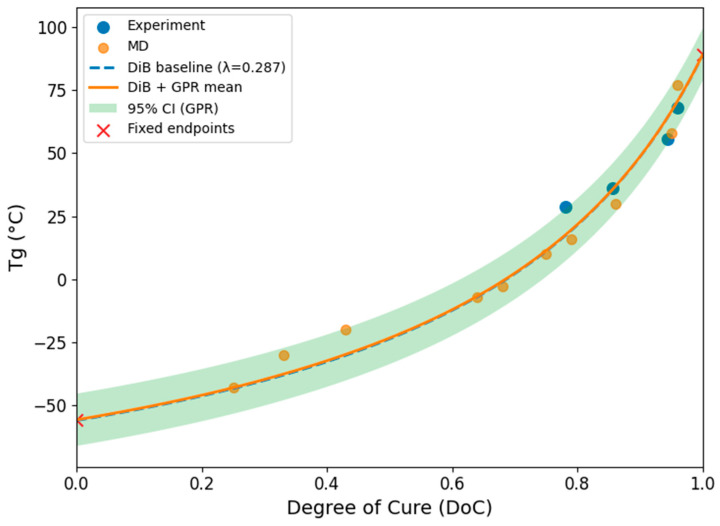
Predictions from the DiBenedetto model for PU augmented with GPR.

To fit the predictive model, in total, 16 data points were combined from experimental and MD data, a cross-validation was performed by the k-fold cross-validation method (*k* = 5), and the fitted curve is plotted as the blue dashed line in Figure 9. The noise-based weighting controls both the data influence on the mean prediction and the width of the confidence interval. The best globally fitted *λ* = 0.287 was selected by cross-validation with the lowest RMSE in the defined region. This model captures the globally monotonic increasing behavior of *T_g_* with *DoC* and strictly satisfies the predefined physical endpoints at *DoC* = 0 and 1.

By superimposing GP residuals onto the DiBenedetto baseline, the proposed model (solid orange curve) effectively corrects these local deviations (systematic and random noises) while preserving the physically meaningful trends imposed by the baseline and hard boundary constraints. The GPR correction primarily acted on regions where experimental and MD data persistently deviate from the baseline between 0.3 and 0.6, thereby enhancing data consistency across the entire *DoC* range without introducing non-physical oscillations. Notably, the model avoids overfitting sparse regions of the dataset, as evidenced by the final curve’s smoothness and the absence of abnormal curvature near the endpoints. The wider 95% confidence interval bands observed at low and intermediate degrees of cure arise from sparse experimental measurements and the relatively higher noise assigned to the MD data, where the automatically selected noise levels were 0.4 for MD and 0.4 for experiments. The predicted curve captures the theory-guided DiBenedetto trend well, and the model incorporates a reliable treatment of measurement and simulation noise.

This proposed surrogate model provides a practical link between molecular-scale curing behavior and process-level optimization. By enabling rapid prediction of *T_g_* evolution as a function of *DoC*, the model can be coupled with thermal–kinetic simulations to estimate viscosity and modulus development during manufacturing. For pultrusion, it informs die temperature zoning, pulling speed, and residence time selection to improve cure uniformity and reduce residual stresses. For injection molding, this supports optimization of mold temperature, holding time, and cooling schedules to avoid premature solidification or incomplete curing. Replacing computationally expensive molecular simulations with a physics-informed surrogate further enables fast parametric studies, offering a pathway toward digital twin-assisted process tuning.

## 4. Conclusions

In this study, the curing behavior of the PU system was characterized using thermal and rheological measurements conducted by DSC and a rheometer. The apparent *E_a_* determined using the isoconversional method was 46.12 kJ/mol. A KS curing kinetics model was established to describe the curing evolution. In addition, the gelation point was identified based on combined DSC and rheological measurements, corresponding to a *DoC* of approximately 0.53.

Due to the rapid curing behavior of the PU system, *T_g_* was determined using a combination of experimental measurements and MD simulations. At the molecular scale, MD simulations were performed using LAMMPS, from which the corresponding *T_g_* values were extracted at different *DoC*. GPR was employed as a bridging framework to integrate experimental DSC data and MD results, with cross-validation used to ensure predictive robustness. A topological crosslinking approach was implemented in LAMMPS under different temperature conditions, from which a global DiBenedetto parameter of λ = 0.29 was determined. GPR enabled reliable forecasting of the trend of *T_g_*–*α* combined MD and experimental results, with CI regions indicating *DoC* ranges dominated by MD data and limited experimental constraints. Furthermore, GP residuals effectively capture systematic, physics-driven biases in the *T_g_* relationship, biases that cannot be fully described by the DiBenedetto model alone.

## Figures and Tables

**Table 1:** Curing kinetics of PU derived from DSC measurements conducted at 3, 5, 10, and 20 °C/min.

Heating Rate, β	*T_onset_* (°C)	*T_p_* (°C)	*T_endset_* (°C)	Curing Duration (min)	∆*H* (J/g)
3 °C/min	12.70	59.12	122.81	35.12	108.34
5 °C/min	20.39	67.50	183.53	25.17	112.28
10 °C/min	40.74	84.60	169.89	13.81	149.64
20 °C/min	37.37	93.80	142.42	7.40	119.63

**Table 2:** Critical curing parameters of PU determined from DSC at heating rates of 3, 5, 10, and 20 °C/min.

Heating Rate	*α_p_*	*α_M_*	*α_p_* ^∞^
3 °C/min	0.325	0.201	0.421
5 °C/min	0.381	0.200	0.448
10 °C/min	0.407	0.200	0.450
20 °C/min	0.389	0.198	0.450

**Table 3:** Correlation (R^2^) between degree of cure and curing rate at heating rates of 3, 5, and 10 °C/min.

Heating Rate	*R* ^2^	
	*DOC*	Curing Rate
3 °C/min	0.997	0.953
5 °C/min	0.999	0.972
10 °C/min	0.999	0.981

**Table 4:** Critical values of PU evaluated from DSC recorded at 3, 5, and 10 °C/min heating rates.

Resin	*A*_1_	*A*_2_	*m*	*n*	*E_a_* (kJ/mol)	*R* ^2^
PU	1.002e-2	3.516e4	0.110	1.6384	46.12	0.97

## Data Availability

The original contributions presented in this study are included in the article. Further inquiries can be directed to the corresponding author.

## Abbreviations

The following abbreviations are used in this manuscript:

PU	Polyurethane
*T_g_*	Glass transition temperature
DSC	D ifferential scanning calorimetry
TMA	T hermomechanical analysis
*DoC*	D egree of cure
MD	Molecular dynamics
EMC	Enhanced Monte Carlo
ML	M achine learning
GPR	Gaussian process regression
CTE	Coefficient of thermal expansion
OPLS-AA	Optimized potentials for liquid simulation all-atom
COMPASS	Condensed-phase Optimized Molecular Potential for Atomistic Simulation Studies
PCFF	Polymer consistent force field
NPT	Constant number of particles, pressure, and temperature
*RMSE*	Root mean square error
*KS*	Kamal–Sourour
*TTT*	Time–temperature–transformation
